# β-caryophyllene, an FDA-Approved Food Additive, Inhibits Methamphetamine-Taking and Methamphetamine-Seeking Behaviors Possibly *via* CB2 and Non-CB2 Receptor Mechanisms

**DOI:** 10.3389/fphar.2021.722476

**Published:** 2021-09-09

**Authors:** Xiang-Hu He, Ewa Galaj, Guo-Hua Bi, Yi He, Briana Hempel, Yan-Lin Wang, Eliot L. Gardner, Zheng-Xiong Xi

**Affiliations:** ^1^ Molecular Targets and Medications Discovery Branch, National Institute on Drug Abuse, Intramural Research Program, Baltimore, MD, United States; ^2^ Department of Anesthesiology, Zhongnan Hospital of Wuhan University, Hubei, China

**Keywords:** β-caryophyllene, dopamine, CB2 receptor, methamphetamine, self-administration, reinstatement

## Abstract

Recent research indicates that brain cannabinoid CB2 receptors are involved in drug reward and addiction. However, it is unclear whether β-caryophyllene (BCP), a natural product with a CB2 receptor agonist profile, has therapeutic effects on methamphetamine (METH) abuse and dependence. In this study, we used animal models of self-administration, electrical brain-stimulation reward (BSR) and *in vivo* microdialysis to explore the effects of BCP on METH-taking and METH-seeking behavior. We found that systemic administration of BCP dose-dependently inhibited METH self-administration under both fixed-ratio and progressive-ratio reinforcement schedules in rats, indicating that BCP reduces METH reward, METH intake, and incentive motivation to seek and take METH. The attenuating effects of BCP were partially blocked by AM 630, a selective CB2 receptor antagonist. Genetic deletion of CB2 receptors in CB2-knockout (CB2-KO) mice also blocked low dose BCP-induced reduction in METH self-administration, suggesting possible involvement of a CB2 receptor mechanism. However, at high doses, BCP produced a reduction in METH self-administration in CB2-KO mice in a manner similar as in WT mice, suggesting that non-CB2 receptor mechanisms underlie high dose BCP-produced effects. In addition, BCP dose-dependently attenuated METH-enhanced electrical BSR and inhibited METH-primed and cue-induced reinstatement of drug-seeking in rats. *In vivo* microdialysis assays indicated that BCP alone did not produce a significant reduction in extracellular dopamine (DA) in the nucleus accumbens (NAc), while BCP pretreatment significantly reduced METH-induced increases in extracellular NAc DA in a dose-dependent manner, suggesting a DA-dependent mechanism involved in BCP action. Together, the present findings suggest that BCP might be a promising therapeutic candidate for the treatment of METH use disorder.

## Introduction

Methamphetamine (METH) is one of the most addictive psychostimulants. Following cannabis, it is the second most widely abused illicit drug worldwide–possibly due to its widespread availability and relatively low costs (Brensilver et al., 2013; Panenka et al., 2013; Rawson, 2013). METH abuse produces serious social and public health problems worldwide (Vearrier et al., 2012; Courtney and Ray, 2014). A number of therapeutic ligands such as methylphenidate (Miles et al., 2013), modafinil (Shearer et al., 2009), topiramate (Johnson et al., 2007), aripiprazole (Newton et al., 2008) and sertraline (Zorick et al., 2011) have been evaluated in clinical trials for the treatment of METH use disorder (Ling et al., 2006). However, to date, no effective medications have been approved by the United States Food and Drug Administration (FDA) for the treatment of METH addiction (Brackins et al., 2011; Rawson, 2013).

Accumulating evidence indicates that the endocannabinoid system in the brain is involved in the rewarding effects of drugs of abuse (Covey et al., 2015; Zlebnik and Cheer, 2016; Galaj and Xi, 2019; Jordan et al., 2020). The endocannabinoid system consists of cannabinoid receptors, endogenous ligands and enzymes (Di Marzo, 2009; Galaj and Xi, 2020). To date, both CB1 and CB2 receptors have been cloned and identified as G-protein-coupled receptors (Svízenská et al., 2008). Early studies have mainly focused on brain CB1 receptors, because CB1 receptors are highly expressed in the central nervous system (Wilson and Nicoll, 2002; Wilson and Nicoll, 2002; Iversen, 2003). Indeed, numerous studies have demonstrated that CB1 receptors play a vital role in drug reward and addiction. Some cannabinoid CB1 receptor antagonists have been tested against the effects of cocaine (Gobira et al., 2019), heroin (Solinas et al., 2003; Navarro et al., 2004), METH (Vinklerová et al., 2002; Schindler et al., 2010; Rodriguez et al., 2011), and nicotine (Shoaib, 2008) in animal models. However, clinical trials with rimonabant, a selective CB1 receptor antagonist or inverse agonist, failed due to severe unwanted side-effects such as depression and suicidal tendency (Le Foll et al., 2009).

In addition to the CB1 receptor, recent studies indicate that CB2 receptors are also expressed in brain regions related to drug abuse and addiction (Gong et al., 2006; Svízenská et al., 2008; Zhang et al., 2014, 2015). CB2 receptors have been found to modulate cocaine self-administration (Aracil-Fernández et al., 2012; Galaj et al., 2020a; Jordan et al., 2020) and cocaine- or nicotine-induced conditioned place preference (Ignatowska-Jankowska et al., 2013). Our previous study found that JWH 133, a selective CB2 receptor agonist, dose-dependently inhibits intravenous cocaine self-administration and this effect is blocked by AM630, a selective CB2 receptor antagonist, and is absent in CB2-KO mice (Xi et al., 2011). These findings suggest that brain CB2 receptors might be a new target in medication development for the treatment of substance use disorders.

(E)-β-caryophyllene (BCP) is a common constituent of essential oils in numerous spice and food plants and a major component in the cannabis sativa plant (Mediavilla and Steinemann, 1997; Sharma et al., 2016). Due to its distinctive flavor and an excellent safety profile, BCP has been approved by the FDA as a “generally recognized as safe” food or cosmetic additive (CFR - Code of Federal Regulations Title 21, 2020). BCP was first synthesized in 1964 (Corey et al., 1964) and later identified as a selective agonist of CB_2_ receptors (*K*_i_ = 155 nM) with ∼60-fold selectivity for CB2 over CB1 receptor (Ki > 10 μM) (Gertsch et al., 2008). BCP has been shown to exhibit its anti-inflammatory, antioxidant, antiviral, and analgesic effects (Cho et al., 2007; Gertsch et al., 2008; Katsuyama et al., 2013; Chicca et al., 2014; Guo et al., 2014; Klauke et al., 2014; Fidyt et al., 2016). Recently, BCP has been found to confer protection against various diseases, including cerebral ischemic injury (Chang et al., 2013), anxiety and depressive disorders (Bahi et al., 2014), alcohol use disorder (Al Mansouri et al., 2014), nicotine dependence (He et al., 2020) and cocaine abuse (Galaj et al., 2021). However, it is unknown whether BCP is also effective against METH reward, intake, and relapse.

Therefore, in the present study, we investigated: 1) whether BCP treatment can inhibit METH self-administration under both fixed-ratio 2 (FR2) and progressive-ratio (PR) schedules of reinforcement in rats; 2) whether deletion of CB2 receptors in CB2-knockout (CB2-KO) mice prevents BCP action on METH self-administration; 3) whether BCP can block METH action on electrical brain-stimulation reward in rats; 4) whether BCP can reduce METH- or cue-induced reinstatement of drug seeking; and 5) whether a dopamine-dependent mechanism is involved in BCP’s potential therapeutic effects against METH-taking and METH-seeking behavior, as assessed by *in vivo* microdialysis.

## Materials and Methods

### Animals

Male Long–Evans rats (Charles River Laboratories, Raleigh, NC) were used in all experiments. Wild-type (WT) and CB2-KO mice with C57BL/6J genetic backgrounds were used only in METH self-administration experiment to determine whether a CB2 receptor-dependent mechanism underlies BCP action. WT and CB2-KO mice (Buckley et al., 2000) were bred within the Transgenic Animal Breeding Facility of the National Institute on Drug Abuse (NIDA). All animals were housed individually in a climate-controlled animal room on a reversed light–dark cycle with free access to food and water. All experimental procedures were carried out in accordance with the *Guide for the Care and Use of Laboratory Animals* of the United States National Research Council and were approved by the NIDA Animal Care and Use Committee.

### Drugs and Chemicals

Methamphetamine HCl (METH) was provided by the Research Pharmacy of the National Institute on Drug Abuse Intramural Research Program and dissolved in sterile 0.9% physiological saline. BCP was obtained from MilliporeSigma (Burlington, MA, United States) and dissolved in 5% Kolliphor EL (i.e., Cremophor) (BASF Pharma, Ludwigshafen, Germany). The BCP doses were chosen from our previous reports (Galaj and Xi, 2020; He et al., 2020). AM630 was purchased from Tocris Division of Bio-Techne (Minneapolis, MN, United States) and dissolved in saline; the doses of AM630 (3, 10 mg/kg) were chosen based on our previous experiments (Galaj and Xi, 2020).

### Surgery

Under standard aseptic surgical techniques, all animals were prepared for experimentation by surgical catheterization of the right external jugular vein as described by Xi et al. (Xi et al., 2011; Galaj et al., 2020b). After all animals were anesthetized by an intraperitoneal injection of sodium pentobarbital (65 mg/kg, i.p.), a microrenathane catheter (Braintree Scientific Inc., Braintree, MA, United States) was inserted into the right jugular vein. After being sutured into place, the catheter was passed subcutaneously to the top of the skull and exited into a connector (a modified 24 g cannula; Plastics One, Roanoke, VA, United States), then mounted to the skull with jeweler’s screws and dental acrylic. To prevent clogging, the catheters were flushed daily with a gentamicin-heparin-saline solution (30 IU/ml heparin) (ICN Biochemicals, Cleveland, OH, United States).

### Apparatus

The intravenous self-administration experiments were conducted in operant chambers (32 × 25 × 33 cm) from MED Associates Inc. (Georgia, VT, United States). Each chamber contained two levers: one active and one inactive, located 6.5 cm above the floor. A cue light and a speaker were located 12 cm above the active lever. The house light was turned on during each 3 h test session. To facilitate acquisition and maintenance of drug self-administration behavior, each drug infusion was paired with a conditioned cue light and a cue sound (tone). Each press of the active lever activated the infusion pump; presses of the inactive lever were counted but had no consequences.

### General Procedure

After recovery from surgery, animals were placed into standard operant chambers for drug self-administration under a fixed ratio 1 (FR1) reinforcement schedule. Each session lasted 3 h during which active lever presses produced delivery of i.v. METH (0.05 mg/kg/infusion) in a volume of 0.08 ml over 4.6 s. During the 4.6 s infusion time, additional responses on the active lever were recorded but did not lead to additional infusions. Inactive lever presses were counted but had no consequence. After a stable pattern of self-administration was established, rats were then randomly assigned to one of the following three groups: 1) METH self-administration under an FR2 schedule of reinforcement in rats; 2) METH self-administration under a PR schedule of reinforcement in rats; 3) METH self-administration under a FR2 schedule of reinforcement followed by extinction (or forced abstinence) and reinstatement tests. As described previously (Xi et al., 2008, 2011; Zhang et al., 2014), in all experiments, BCP was given (i.p.) 30 min prior to testing. The CB2 antagonist AM 630 was administrated (i.p.) 30 min prior to the injection of BCP.

### Experiment 1. Methamphetamine Self-Administration Under a Fixed-Ratio 2 Reinforcement Schedule in Rats

After transition from a FR1 to FR2 schedule of reinforcement, drug self-administration training continued with METH (0.05 mg/kg/infusion). The following criteria were used to assess whether stable drug-maintained responding was established: less than 10% variability in intra-session responding and less than 10% variability in the number of active lever presses for at least three consecutive days. To prevent drug overdose, each animal was limited to a maximum of 50 infusions per self-administration session. After stable rates of responding were established, each subject randomly received one of four doses of BCP (10, 25, 50, 100 mg/kg, i.p.), or vehicle (equal volume of 5% Kolliphor solution) 30 min prior to the test session. For subjects that received pretreatment with the CB2 antagonist AM630, the antagonist (3 or 10 mg/kg, i.p.) was administrated 30 min prior to BCP. Animals then received an additional 5–7 days of self-administration of METH alone until a baseline response rate was reestablished prior to being tested with another dose of BCP. The order of testing with different doses of BCP or AM 630 was counterbalanced.

### Experiment 2. Methamphetamine Self-Administration Under a Progressive-Ratio Reinforcement Schedule in Rats

After stable METH self-administration under a FR2 schedule of reinforcement was established, an additional group of rats were switched to METH self-administration (0.05 mg/kg/infusion) under a progressive-ratio (PR) schedule, during which the lever-pressing work requirement needed to receive a single i.v. METH infusion was progressively raised within each test session [see details in (Richardson and Roberts, 1996)] according to the following PR series: 1, 2, 4, 6, 9, 12, 15, 20, 25, 32, 40, 50, 62, 77, 95, 118, 145, 178, 219, 268, 328, 402, 492, and 603 until a break point was reached. The break point was defined as the maximum number of lever presses completed for the last METH infusion prior to a 1-h period during which no infusions were obtained. Animals self-administered METH daily under the PR reinforcement conditions until day-to-day variability in break points fell within 1-2 ratio increments for three consecutive days. After a stable break point was established, subjects were assigned to different subgroups to determine the effects of BCP (10, 25, 50 mg/kg, i.p.) or vehicle (equal volume of 5% Kolliphor solution) on PR break point for METH self-administration. To evaluate the mechanism by which BCP produces its effects on METH self-administration, AM630, a CB2 antagonist (3 mg/kg) was administrated 30 min prior to the injection of BCP. Since it is relatively difficult to re-establish a stable break point level after each drug test, we used a between-subjects design rather than a within-subjects design to determine the dose–response effects of BCP and BCP plus AM 630 on break point for METH.

### Experiment 3: Locomotor Activity in Rats

Three groups of rats were used to observe the effects of BCP on spontaneous locomotor activity. On the test day, rats were initially placed in locomotor detection chambers (Accuscan, Columbus, OH, United States) for a 30-min habituation period, and then each rat was administered one of the two doses of BCP (25, 50 mg/kg, i.p.) or vehicle (5% Kolliphor solution). The habituation was chosen because animal locomotor activity within the initial 30 min in locomotor chamber is high and variable, and therefore, we chose to observe the locomotor effects of BCP after basal level of locomotion stabilized. After the BCP injection, rats were placed back into the locomotor chambers for 2 h to record possible alterations in locomotion. Total distance was used to evaluate the effects of BCP on locomotion.

### Experiment 4. Methamphetamine Self-Administration in Wild-Type and CB2-Knockout Mice

To further examine possible involvement of a CB2 receptor mechanism in BCP’s action, we used CB2-KO mice as controls (*n* = 8) and their WT littermates (*n* = 9) in a self-administration paradigm. Briefly, animals were trained to self-administer METH (0.05 mg/kg, i.v.) under an FR1 schedule of reinforcement during daily 3-h sessions for approximately 2–3 weeks. Responding on the active lever activated the syringe pump–producing an i.v. infusion of METH (0.015 ml) and presentation of the light cue above the active lever and the tone cue. Responses on the inactive lever were counted but had no consequences. During the 4.2-s infusion period, additional responses on the active lever were recorded but did not lead to additional infusions. Animals were tested with BCP (0, 25, 50, 100 mg/kg i.p., 30 min prior to the test session) after stable METH self-administration was achieved, defined as 1) at least 20 METH infusions during the 3-h session, 2) less than 20% variability in daily METH infusions across two consecutive days, and 3) an active/inactive lever press ratio exceeding 2:1. Mice then received an additional 5–7 days of METH self-administration between BCP tests until stable self-administration was re-established as described above. The order of BCP doses was counterbalanced.

### Experiment 5: Methamphetamine-Induced Reinstatement of Drug Seeking in Rats

After stable METH self-administration training, a third group of rats was exposed to extinction conditions, during which METH was replaced by saline, and the METH-associated cue light and tone were turned off. Daily extinction sessions continued until lever pressing was <10 per 3 h session for three consecutive days. Then, rats were divided into three BCP dose groups. On the reinstatement test day, each group received either vehicle (5% Kolliphor solution) or one of the BCP doses (25, 50 mg/kg). Thirty min later, rats were given a priming injection of METH (1 mg/kg, i.p.) and immediately tested in a reinstatement test. During the reinstatement test, which lasted 3 h, lever-pressing responses did not lead to either METH infusions or presentation of the conditioned cues. METH-induced lever-pressing responses were recorded. This priming dose of METH was found to produce robust reinstatement of METH seeking in our previous studies (Higley et al., 2011).

### Experiment 6: Cue-Induced Methamphetamine Seeking in Rats

Additional groups of rats were used to assess the effects of BCP pretreatment on contextual cue-induced METH-seeking behavior. This “incubation of craving” model was chosen because it mimics relapse in humans after forced abstinence (Altshuler et al., 2021). In addition, we have found over many years of experience that contextual cue-induced drug seeking is more robust than classical cue-induced reinstatement responding, and therefore, it is a more sensitive measure of cue-induced changes in drug-seeking behavior. After stable METH self-administration was achieved under a FR2 schedule of reinforcement, rats underwent forced abstinence in their home cages. After 21 days of withdrawal from METH self-administration, rats were divided into four experimental groups; each group received either vehicle (5% Kolliphor solution) or one of the three doses of BCP (25, 50, 100 mg/kg). 30 min after the injection on the test day, the rats were re-placed into the same self-administration chambers. Contextual cue-induced drug seeking was conducted under conditions identical to that of self-administration, except that responses on the active lever (under a FR2 schedule) resulted in contingent presentation of the cues without METH availability (no infusions). Responses on the inactive lever were recorded but had no programmed consequences. Each reinstatement test lasted for 3 h.

### Experiment 7: Electrical Brain Stimulation Reward in Rats

We then assessed the effects of BCP on METH-enhanced electrical brain-stimulation reward (BSR). The procedures of electrical BSR were the same as we reported recently (Spiller et al., 2019). Briefly, lever pressing for electrical BSR was reinforced by a stimulation current at different frequencies from 141 to 25 Hz in a decreasing series of 16 discrete 0.05 log steps. At each pulse frequency, there were two 30-s trials, each followed by lever retraction for 5 s. A response rate for each frequency was defined as the mean number of lever responses during two 30-s trials. The BSR threshold (*θ*_0_) was defined as the minimum frequency at which an animal responded for stimulation, calculated using the Gompertz sigmoidal model (Coulombe and Miliaressis, 1987). In addition, Ymax was measured as maximum number of lever presses. The testing phase began once stable BSR responding was achieved (<20% variation in *θ*_0_ over three consecutive days). On the test day rats received systemic injection of BCP (0, 50, or 100 mg/kg) 30 min prior to METH injection (2 mg/kg) and later were allowed to lever-press for brain-stimulation. After each test, a new baseline *θ*_0_ was established and rats were re-tested with a different dose of BCP in the presence of METH treatment. The BCP effects on BSR were also evaluated in the absence of METH.

### Experiment 8: *In vivo* Brain Microdialysis in Rats

Microdialysis experiments were performed in six additional groups of rats to evaluate the effects of vehicle (5% Kolliphor solution) or BCP (25, 50 mg/kg) alone on basal levels of extracellular DA or BCP pretreatment on METH-enhanced NAc dopamine (DA). Microdialysis protocols and probe construction were as reported previously (Xi et al., 2006). Guide cannulae (20 gauge; Plastics One, Roanoke,VA) were surgically implanted into the NAc (anteroposterior, +1.6 mm; mediolateral, ±1.8 mm; dorsoventral,—4.3 mm, angled 6° from vertical) using standard surgical and stereotaxic techniques. Microdialysis probes were inserted into the NAc 12 h before the experiment to minimize damage-induced neurotransmitter release. During the experiment, microdialysis buffer was perfused through the probe (2.0 ml/min) for at least 2 h before sampling started. Samples were collected every 20 min into 10 μl of 0.5 M perchloric acid to prevent neurotransmitter degradation. After 1 h baseline collection, one of the two doses of BCP (25, 50 mg/kg, i.p.) or vehicle (5% Kolliphor solution) were administered 40 min prior to METH administration. All samples were frozen at 80°C until analyzed. After microdialysis experiments were completed, rats were anesthetized with a high dose of pentobarbital (>100 mg/kg i.p.) and perfused transcardially with 0.9% saline followed by 10% formalin. Brains were removed and placed in 10% formalin for histological verification of microdialysis probe locations in rat brain.

Microdialysate DA was measured by high performance liquid chromatography (HPLC) with an ESA (ESA Biosciences, Chelmsford, MA) electrochemical (EC) detection system as described previously (Xi et al., 2006), upgraded by a Coulochem III EC detector. Areas under the curve (AUC) for DA were measured and quantified with external standard curves. The minimum detection limit for DA was 1–10 fmol.

### Data Analysis

All data are presented as means ± SEM. Separate one-way analyses of variance (ANOVAs) were used to analyze the effects of BCP on drug self-administration, methamphetamine or cue-induced reinstatement, NAc DA and locomotion. A two-way ANOVA with time as the repeated measure was used to analyze the effects of BCP on METH self-administration in WT and CB2-KO mice and on NAc DA. The Student–Newman–Keuls post-hoc test or Tukey’s honestly significant difference (HSD) test was used for multiple group comparisons. The statistical significance was set at a probability level of *p* < 0.05 for all tests.

## Results

### β-Caryophyllene Attenuates Methamphetamine Self-Administration Under a Fixed-Ratio 2 Schedule of Reinforcement

**FIGURE 1:**
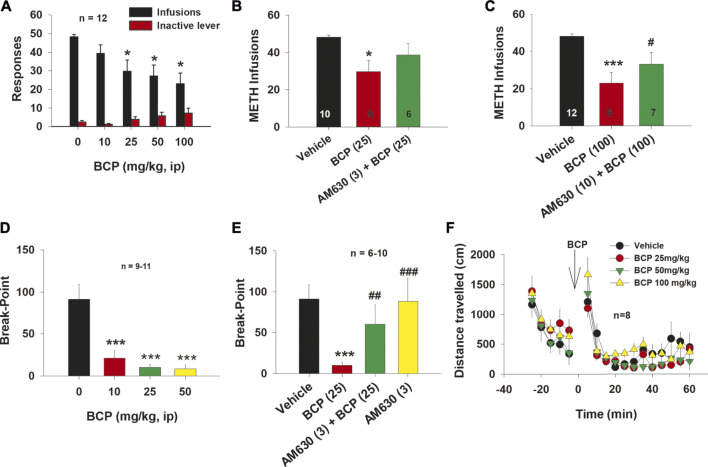
The effect of BCP on METH self-administration under FR2 and progressive-ratio reinforcement schedules in rats. **(A)**: Administration of BCP dose-dependently decreased the number of METH self-infusions. **(B):** Pretreatment with AM630 (3 mg/kg) blocked BCP (25 mg/kg)-induced reduction in METH self-administration. **(C)**: Pretreatment with AM630 (10 mg/kg) also blocked the attenuating effects of BCP (100 mg/kg) on METH self-administration. **(D)**: BCP dose-dependently reduced the break-point level for METH self-administration under PR reinforcement conditions. **(E)**: Pretreatment with AM630 (3 mg/kg) blocked BCP’s action under PR reinforcement conditions (25 mg/kg), while AM630 (3 mg/kg) alone failed to alter the break-point for METH self-administration. **(F)**: BCP, at 25, 50 and 100 mg/kg, did not alter open-field locomotor activity in rats. Data are presented as means ± SEM. *p < 0.05, ***p < 0.001, when compared to the vehicle group. ^#^ *p* < 0.05, ^##^ *p* < 0.01; ^###^ *p* < 0.001, compared to “BCP (25)” or “BCP (100)” groups.

Figure 1Ashows the effects of BCP on METH self-administration under a FR2 reinforcement schedule. Treatment with BCP dose-dependently inhibited METH self-administration. A two-way ANOVA with repeated measurements over BCP doses revealed a statistically significant infusion *vs* inactive lever response main effect (*F*_1, 11_ = 126.92, *p* < 0.001) and, most relevantly, a significant interaction effect between BCP dose and infusion vs inactive lever responding (*F*_4,42_ = 6.41, *p* < 0.001). Post-hoc tests revealed a statistically significant reduction in METH self-administration after 25 mg/kg (*q* = 5.26, *p* < 0.001), 50 mg/kg (*q* = 5.99, *p* < 0.001) or 100 mg/kg (*q* = 7.17, *p* < 0.001), but not after 10 mg/kg (*q* = 2.54, *p* = NS) BCP, when compared to the vehicle group.

To explore a potential role of CB2 receptors in BCP’s action on METH self-administration, we administered the CB2 receptor antagonist AM630 (3 mg/kg, i.p.) 30 min prior to BCP treatment. As shown in Figure 1B, pretreatment with AM630 blocked the inhibitory effects of 25 mg/kg BCP on METH self-administration (F_2, 27_ = 4.57, *p* < 0.05). Post-hoc tests revealed a statistically significant reduction in METH infusions after 25 mg/kg (*q* = 4.27, *p* < 0.05), but not after 25 mg/kg BCP plus 3 mg/kg AM630 (*q* = 1.81, *p* > 0.05), as compared to the vehicle control group.

Similarly, pretreatment with AM 630 (10 mg/kg, i.p.) also reversed the inhibitory effects of 100 mg/kg BCP on METH self-administration (Figure 1C, F_2, 28_ = 8.84, *p* < 0.001). Post-hoc tests revealed a statistically significant reduction in METH infusions after 100 mg/kg (*q* = 5.88, *p* < 0.001), but not after 100 mg/kg BCP plus 10 mg/kg AM630 (*q* = 1.74, *p* > 0.05), when compared to the vehicle treatment group.

### β-Caryophyllene Reduces Progressive-Ratio Break-point Level for Methamphetamine Self-Administration

Figure 1Dshows that treatment with BCP (10, 25, 50 mg/kg, i.p.) dose-dependently shifted the PR break-point for METH self-administration downward (*F*_3, 35_ = 14.93, *p* < 0.001). Post-hoc between group comparisons revealed a significant reduction in break-point for METH self-administration after 10 mg/kg (*q* = 6.69, *p* < 0.001), 25 mg/kg (*q* = 7.74, *p* < 0.001) or 50 mg/kg (*q* = 8.32, *p* < 0.001) BCP treatment, as compared to the vehicle treatment group.

As shown in Figure 1E, pretreatment with AM630 blocked the effects of BCP (25 mg/kg) on the PR break-point for METH self-administration (*F*_3, 27_ = 4.76, *p* < 0.01). Post-hoc tests revealed a statistically significant reduction in break-point after 25 mg/kg BCP (*q* = 4.91, *p* < 0.01), but not after 25 mg/kg BCP plus 3 mg/kg AM630 (*q* = 1.68, *p* > 0.05) or 3 mg/kg AM630 alone (*q* = 0.14, *p* > 0.05), when compared with the vehicle treatment group.

To determine whether the reduction in METH self-administration was due to BCP-induced sedation or locomotor impairment, we evaluated the effect of BCP on open field locomotion in rats. Figure 1F shows that BCP, at the same doses, failed to alter open-field locomotion. A two-way ANOVA with BCP treatment and time as repeated-measures factors revealed a statistically significant main effect of time (*F*_17,255_ = 54.716, *p* < 0.05) but no main effect of BCP treatment (F_3,45_ = 0.929; *p* < 0.43) or time × treatment interaction (F_51,765_ = 1.00; *p* = 0.465).

### β-Caryophyllene Reduces Methamphetamine Self-Administration in Wild-Type Mice and at a High Dose in CB2-Knockout Mice

**FIGURE 2:**
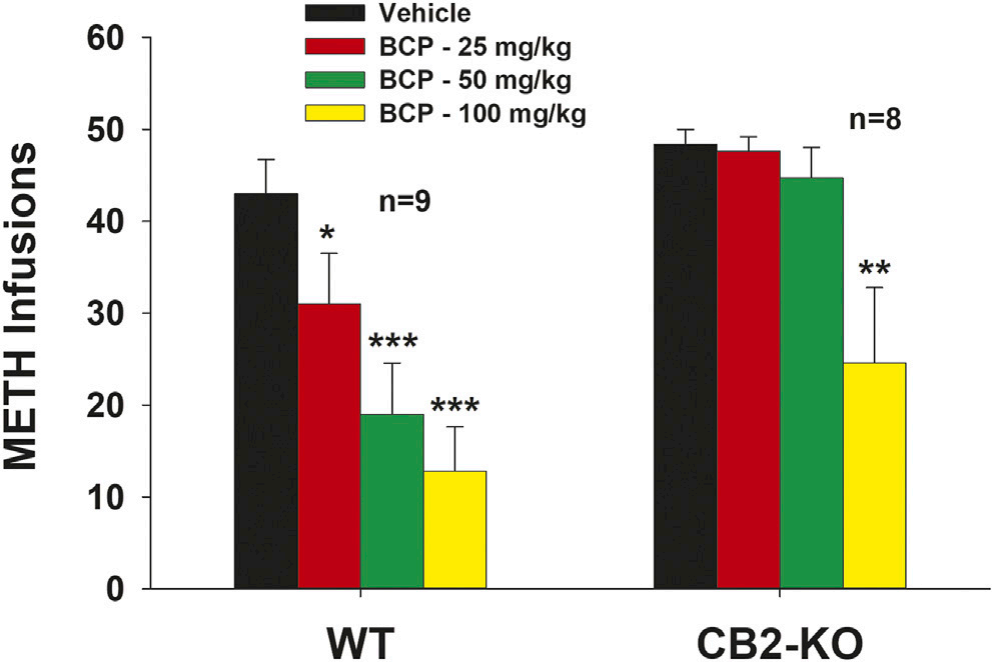
The effects of BCP on METH self-administration under a fixed-ratio 1 (FR1) reinforcement schedule in WT and CB2-KO mice. BCP dose-dependently decreased METH self-administration in WT mice, while only at the very high dose of 100 mg/kg, BCP inhibited METH self-administration in CB2-KO mice. *p < 0.05, **p < 0.01, ***p < 0.001, when compared to the vehicle group.

To further assess the potential involvement of CB2 receptors in the inhibitory effects of BCP on METH self-administration, we used transgenic mice lacking CB2 receptors. WT and CB2-KO mice were trained to self-administer METH under FR1 reinforcement. Systemic administration of BCP dose-dependently inhibited METH self-administration in WT mice and CB2-KO mice (Figure 2). A two-way ANOVA with repeated measurements for BCP doses revealed a significant strain (WT vs CB2-KO) main effect (*F*_1, 15_ = 13.29, *p* < 0.01) and BCP dose main effect (*F*_3, 45_ = 15.78, *p* < 0.001), but without strain × BCP interaction (*F*_3, 45_ = 2.17, *p* > 0.05). Post-hoc individual group comparisons revealed a significant reduction in infusions for METH self-administration in WT mice after 25 mg/kg (*q* = 3.00, *p* < 0.05), 50 mg/kg (*q* = 6.00, *p* < 0.001) or 100 mg/kg (*q* = 7.54, *p* < 0.001) BCP, as compared to the vehicle treatment group. Similar post-hoc tests revealed a significant reduction in infusions for METH self-administration in CB2-KO mice after administration of 100 mg/kg (*q* = 5.60, *p* < 0.01), but not after 25 mg/kg (*q* = 0.18, *p* > 0.05) or 50 mg/kg (*q* = 0.85, *p* > 0.05) of BCP, when compared to the vehicle treatment group.

### β-Caryophyllene Reduces Methamphetamine-Enhanced Brain-Stimulation Reward

**FIGURE 3:**
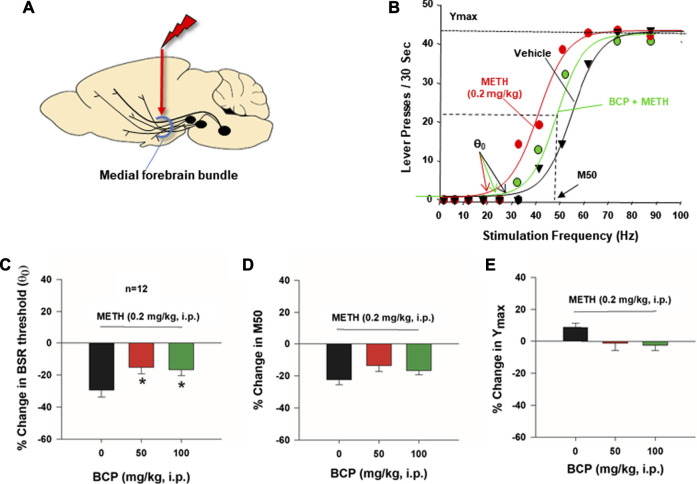
The effects of BCP on METH-enhanced electrical brain-stimulation reward (BSR) in rats (*n* = 12). **(A)**: A diagram showing that electrical stimulation of the medial forebrain bundle at the hypothalamus produces BSR. **(B)**: Representative stimulation–response curves, indicating that METH treatment shifted the stimulation-response curve to the left and decreased the BSR stimulation threshold (θ_0_ value) but not M50; **(C)**: Averaged % changes in BSR stimulation threshold (θ_0_ value), indicating that BCP pretreatment significantly attenuated METH-induced reduction in the θ_0_ value. **(D)**: BCP did not produce a significant reduction in M50. **(E)**: METH and BCP did not produce a significant change in Ymax. *p < 0.05, compared to the vehicle group.

Next, we used the highly sensitive BSR paradigm to shed further light upon the effects of BCP on METH reward. Figure 3A shows the general experimental procedures, in which electrical stimulation was targeted at the medial forebrain bundle at the level for the lateral hypothalamus. Figure 3B shows representative rate-frequency functions for BSR, indicating the BSR stimulation threshold θ0, M50, Ymax, and the effects of METH on BSR in the presence or absence of BCP. METH (0.2 mg/kg, i.p.) significantly decreased the BSR threshold θ_0_ value (i.e., shifted the curve to the left) without affecting asymptotic rates of responding (i.e., no change in Ymax level), indicating that lower stimulation intensity (Hz) was required to produce BSR in the presence of METH, suggesting that METH and rewarding brain stimulation produce an additive or synergistic effect (i.e., that METH enhances BSR). Figure 3C shows that pretreatment with BCP dose-dependently decreased METH-enhanced BSR, as indicated by an increase in BSR stimulation threshold θ_0_ values (F_2,22_ = 5.018; *p* < 0.05). Treatment with BCP did not significantly alter the M50 value (Figure 3D, F_2,22_ = 3.024; *p* > 0.05) or the Ymax level (Figure 3E, F_2,22_ = 3.299; *p* > 0.05). The latter finding concerning Ymax suggests a lack of motoric impairment after BCP and METH administration.

### β-Caryophyllene Reduces Methamphetamine-Primed Reinstatement of Drug Seeking

**FIGURE 4:**
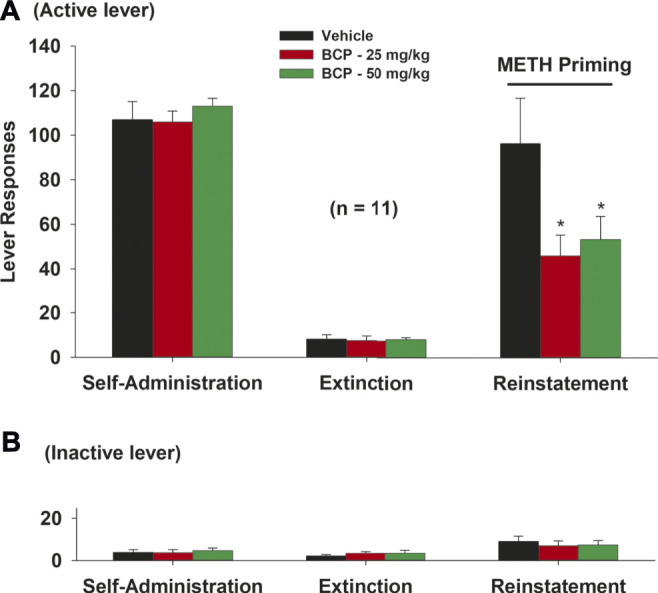
The effects of BCP pretreatment on METH-induced reinstatement of drug seeking in rats. **(A):** Active lever presses during the last session of METH self-administration, last extinction session, and reinstatement test, illustrating that BCP (25, 50 mg/kg, i.p., 30 min prior to test) significantly reduced METH priming-induced reinstatement. **(B)**: BCP, at the same dose, had no effect on inactive lever presses during reinstatement testing. Data are presented as means ± SEM. *p < 0.05, when compared to the vehicle group.

Figure 4 illustrates the total numbers of active and inactive lever presses observed during the last session of METH self-administration, the last session of extinction, and the reinstatement test session in the three different dose groups for BCP (vehicle, 25, 50 mg/kg). A single, non-contingent METH priming injection (1 mg/kg) produced robust reinstatement of extinguished operant responding (i.e., active lever presses) in rats with a history of METH self-administration. Treatment with BCP produced a significant reduction in METH-induced reinstatement of drug-seeking behavior (Figure 4A, active lever responding: *F*_2,30_ = 3.96, *p* < 0.05). Post-hoc tests revealed a significant reduction in METH seeking after 25 mg/kg (*q* = 3.95, *p* < 0.05) or 50 mg/kg (*q* = 3.79, *p* < 0.05) BCP, when compared to the vehicle control group. There were no significant differences in inactive lever responding across BCP dose groups (Figure 4B).

### β-Caryophyllene Attenuates Cue-Induced Methamphetamine Seeking

**FIGURE 5:**
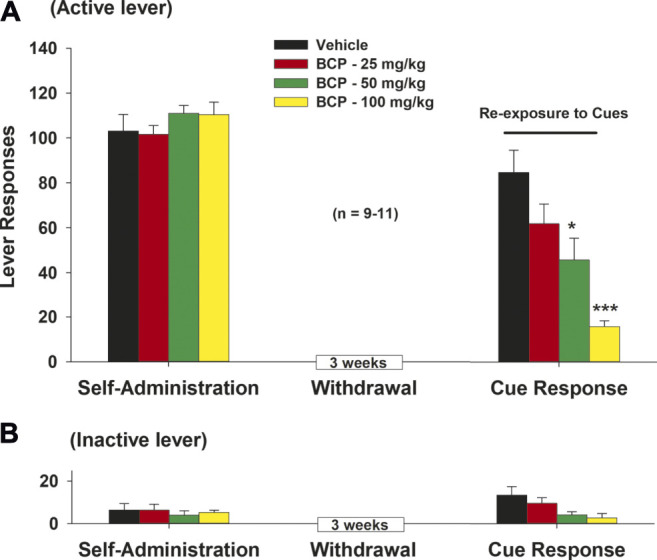
The effects of BCP on METH-associated cue-induced drug seeking in rats after forced abstinence. **(A)**: Systemic administration of BCP (25, 50, 100 mg/kg, i.p., 30 min prior to test) dose-dependently inhibited cue-triggered drug-seeking in rats after 3 weeks of withdrawal from METH self-administration. **(B)**: BCP, at the same doses, failed to alter inactive lever responses during cue exposure test. Data are presented as means ± SEM. *p < 0.05, ***p < 0.001, when compared to the vehicle group.

We also observed the effects of BCP treatment on cue-induced drug seeking in rats after 3 weeks of withdrawal from METH self-administration (e.g., in a forced abstinence craving model). We found that BCP dose-dependently attenuated METH-associated cue-induced drug seeking (Figure 5). A one-way ANOVA of the cue-triggered response data revealed a significant BCP treatment main effect (Figure 5A: *F*_3,36_ = 11.78, *p* < 0.001) on active lever presses. Post-hoc tests revealed that 25 mg/kg (*q* = 3.17, *p* < 0.05), 50 mg/kg (*q* = 4.53, *p* < 0.01) or 100 mg/kg (*q* = 8.29, *p* < 0.001) of BCP significantly reduced active lever responding, when compared to the vehicle control group. There were no significant differences in inactive lever responding across different BCP dose groups (Figure 5B).

### β-Caryophyllene Attenuates Methamphetamine-Enhanced Dopamine in the Nucleus Accumbens

**FIGURE 6:**
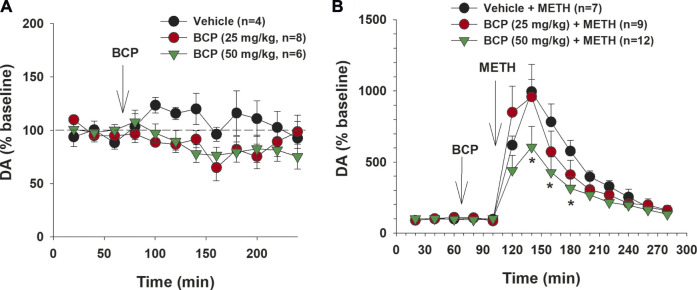
The effects of BCP and METH on extracellular DA in the nucleus accumbens (NAc). **(A)**: BCP, at 25 and 50 mg/kg, failed to produce a significant reduction in extracellular NAc DA. **(B)**: Pretreatment with BCP dose-dependently attenuated METH-induced enhancement of extracellular NAc DA. Data are presented as means ± SEM. *p < 0.05, when compared to the vehicle pretreatment group.

Finally, we examined whether a DA-dependent mechanism might underlie BCP actions against METH by using *in vivo* brain microdialysis. Figure 6A shows that BCP alone, at the doses of 25 or 50 mg/kg, produced no statistically significant effect on extracellular DA in the NAc. A two-way ANOVA with time as the repeated-measures factor revealed a significant main effect of time (F_11, 165_ = 2.09, *p* < 0.05), but did not reveal a BCP treatment main effect (F_2, 15_ = 1.02, *p* > 0.05) or a BCP × time interaction (F_22, 165_ = 1.14, *p* > 0.05), suggesting that BCP alone did not significantly alter NAc DA release. Figure 6B shows that METH (1 mg/kg) in the vehicle pretreatment group caused a rapid and significant increase in extracellular DA level in drug-naive rats, which lasted 2–3 h with a peak effect at 1 h after the injection. Treatment with 50 mg/kg, but not 25 mg/kg, of BCP significantly attenuated the METH-induced increase in extracellular DA. Two-way ANOVAs with time as the repeated-measures factor and BCP dose as the between-subjects factor revealed a significant main effect of time (F_12,300_ = 46.176, *p* < 0.0001) and BCP treatment × time interaction (F_24,300_ = 2.189, *p* < 0.0001), but no main effect of BCP dose (F_2,25_ = 1.599; *p* = 0.22). Post-hoc (Tukey) tests for multiple group comparisons indicated that METH-induced enhancement of extracellular DA was significantly reduced by 50 mg/kg, but not by 25 mg/kg, of BCP, when compared to Veh + METH groups (Figure 6B).

## Discussion

In the present study, we found that systemic administration of the natural CB2R agonist BCP (Gertsch et al., 2008) dose-dependently inhibited intravenous METH self-administration, METH-enhanced brain-stimulation reward, and METH- or cue-induced drug-seeking in rats. Importantly, the inhibitory effects of BCP on METH self-administration were attenuated by the cannabinoid CB2 receptor antagonist AM630, and genetic deletion of CB2 receptors also blocked low dose (25, 50 mg/kg) BCP-induced reduction in METH self-administration, suggesting the possible involvement of CB2 receptor mechanisms. Notably, BCP, at a high dose (100 mg/kg), also inhibited METH self-administration in CB2-KO mice, suggesting that non-CB2 receptor mechanisms are involved in high dose BCP-mediated effects. This is consistent with our previous reports that systemic administration of BCP, at high doses (50, 100 mg/kg), also inhibits cocaine or nicotine self-administration in CB2-KO mice (He et al., 2020; Galaj et al., 2021), suggesting that BCP’s selectivity as a CB2 receptor agonist depends on the BCP dose, and at high doses, it also binds to other (non-CB2) receptors. Furthermore, BCP alone did not produce a significant decrease in extracellular NAc DA, while pretreatment with BCP dose-dependently attenuated METH-induced increase in extracellular DA, suggesting that a DA-dependent mechanism at least in part underlies BCP’s actions against METH.

We and others have previously reported the presence of functional CB2 receptors in the brain, especially in reward-related areas such as the ventral tegmental area (VTA) and the NAc (Gong et al., 2006; Zhang et al., 2014, 2019, 2021a, 2021b; Foster et al., 2016; Jordan and Xi, 2019), suggesting the potential involvement of CB2 receptors in drug abuse. This hypothesis is supported by a number of studies indicating that CB2R agonists or inverse agonists (JWH133, O-1966, Xie2-64, BCP) significantly inhibit cocaine self-administration, cocaine-induced conditioned place preference (CPP), cocaine-induced hyperlocomotion and locomotor sensitization (Xi et al., 2011; Aracil-Fernández et al., 2012; Ignatowska-Jankowska et al., 2013; Zhang et al., 2015; Delis et al., 2017; Jordan et al., 2020; Galaj et al., 2021). Congruently, overexpression of CB2 receptor in the brain also produces anti-cocaine effects (Aracil-Fernández et al., 2012). In addition, BCP, at low doses (10, 25 mg/kg) significantly decreased the break-point for METH self-administration under PR reinforcement, suggesting that BCP has the ability to attenuate animals’ motivation for the drug. The reduction in METH self-administration is unlikely due to non-specific sedative effects or locomotor impairment, because BCP, at the same doses, did not alter basal or cocaine-enhanced locomotor activity (Galaj et al., 2019). The present anti-METH findings are congruent with previous reports that BCP attenuates intravenous cocaine or nicotine self-administration and oral alcohol consumption in rats and mice (Al Mansouri et al., 2014; He et al., 2020; Galaj et al., 2021). They are also congruent with recent reports that a CB2 receptor mechanism mediates the analgesic, anxiolytic and anti-depressant effects of BCP (Bahi et al., 2014; Klauke et al., 2014; Youssef et al., 2019).

We note that the effective doses of BCP that inhibit self-administration of nicotine, cocaine and METH are different. Lower doses (25, 50 mg/kg) of BCP are able to inhibit nicotine (He et al., 2020) or METH self-administration, while a higher dose (100 mg/kg) of BCP is required to inhibit cocaine self-administration, which is not blocked by deletion of the CB2 receptor in CB2-KO mice (Galaj et al., 2020a). This may be related to the reinforcing strength or the doses of drugs of abuse used in those studies. The facts that nicotine is a weak reinforcer compared to cocaine and that the METH dose (0.05 mg/kg/infusion) used in our self-administration experiments is 10-fold lower than the cocaine dose (0.5 mg/kg/infusion) may well explain why BCP, at lower doses, is able to inhibit nicotine or METH, but not cocaine, self-administration, and why genetic deletion of the CB2 receptor in CB2-KO mice is able to prevent low dose, but not high dose, BCP-induced attenuation of drug self-administration, given that BCP at high doses binds to non-CB2 off-targets (Galaj et al., 2020b, but see; Finlay et al., 2020; Santiago et al., 2019).

The precise non-CB2 receptor mechanisms that may be involved remain unclear. We previously reported that genetic deletion and/or pharmacological blockade of the CB1, GRP55, mu opioid, and toll-like receptor 4 (TLR4) failed to alter BCP’s action on cocaine self-administration, suggesting that these receptors are not involved in BCP’s action against cocaine (Galaj et al., 2021). Unexpectedly, we found that peroxisome proliferator-activated receptor-α (PPARα) or PPARγ antagonists dose-dependently attenuated BCP’s action against cocaine self-administration (Galaj et al., 2020a), suggesting that these two receptors may be also involved in BCP’s action against METH. Clearly, more studies are required to test this hypothesis.

It is not fully understood how BCP produces inhibitory effects on METH-taking and METH-seeking behaviors. It is widely believed that the brain CB2 receptor is mainly or exclusively expressed in microglia, not in neurons, and can be upregulated in activated microglia during neuroinflammation (Atwood and Mackie, 2010; López et al., 2018). However, this view is not supported by our findings that neither CB2-immunostaining nor CB2 mRNA was detected in microglia in either normal healthy subjects (Zhang et al., 2014, 2017, 2019) or in mice after acute administration of lipopolysaccharide, an endotoxin that causes severe neuroinflammation and microglia activation (Zhang et al., 2014) or chronic administration of cocaine (Zhang et al., 2017; 2021a). In contrast, we demonstrated clear CB2 receptor expression in multiple phenotypes of neurons, including VTA DA neurons (Zhang et al., 2014, 2017, 2019; Humburg et al., 2021), red nucleus glutamate neurons (Zhang et al., 2021b), and striatal GABA neurons (Zhang et al., 2021a; see a comprehensive review by; Jordan and Xi, 2019). Furthermore, chronic cocaine administration significantly up-regulates CB2 receptor expression in VTA DA neurons and NAc D1 receptor-expressing medium-spiny neurons, not in microglia (Zhang et al., 2014; 2021a). Consistent with these findings, genetic deletion of CB2 receptors from lymphocytes, mainly from monocytes (the precursors of microglial cells), failed to alter JWH133 self-administration (self-medication) to relieve neuropathic pain (Cabañero et al., 2020). In contrast, genetic deletion of CB2 receptor from neurons (syn-Cre X CB2-floxed) significantly altered JWH133 self-administration (Cabañero et al., 2020), suggesting that neuronal CB2 receptor mechanisms underlie the analgesic effects of CB2 receptor activation. However, other work using targeted expression of fluorescent proteins in CB2-reporter mice failed to detect CB2 receptor expression in neurons (Schmöle et al., 2015; López et al., 2018), suggesting that more work is required to further address the role of neuronal *versus* microglial CB2 in BCP action.

**FIGURE 7:**
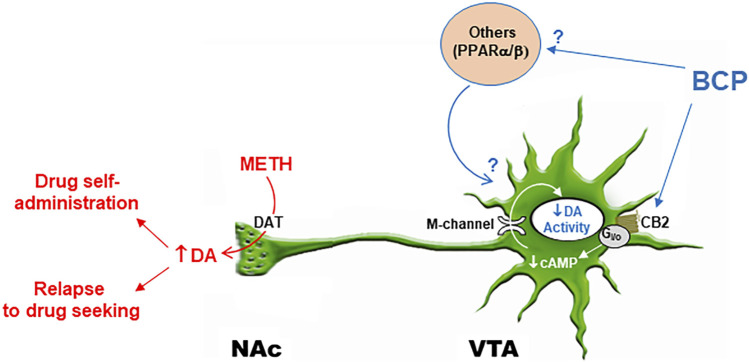
Schematic diagram illustrating possible interaction of BCP and METH in the mesolimbic DA system. METH promotes DA release from presynaptic DA terminals in the NAc and other projection regions via the membrane DA transporter (DAT) and intracellular type 2 vesicular monoamine transports (VMAT2, not shown). BCP binds to CB2 receptors on DA neurons and inhibits DA neuron activity by activation of M-type K^+^ channel via a Gi/o-cAMP-PKA signal pathway (Zhang et al., 2014; Ma et al., 2019), which subsequently attenuates NAc DA response to METH and DA-dependent addiction-related behavior. In addition, BCP may also bind to other non-CB2 receptors such as PPARα/β, which indirectly modulates DA neuron activity.

It is well documented that drug abuse and addiction are closely associated with an increase in extracellular DA in the NAc (Di Chiara and Imperato, 1988; Ranaldi et al., 1999; Le Foll and Goldberg, 2005; Galaj et al., 2019). With respect to the present topic, METH’s highly addictive properties have been attributed to its effect on DA release. METH is a substrate for the dopamine transporter (DAT) and the vesicular monoamine transporter 2 (VMAT2). METH is first taken into the cytoplasm via the DAT and then enters vesicles via the VMAT2. Each molecule of METH that undergoes vesicular entry causes two protons to be extruded, which diminishes vesicular H^+^ concentration. The pH gradient is the main driving force for vesicular loading and retention of DA. In the absence of this pH gradient, DA is rapidly accumulated in the cytoplasm, which reverses the functional direction of the DAT and releases DA into the extracellular space (Elkashef et al., 2008; Freyberg et al., 2016). As noted above, a series of studies have shown that CB2 receptor genes and receptors are expressed in midbrain DA neurons and negatively modulate DA neuronal activity mainly by activation of M-type K^+^ channels (Xi et al., 2011; Zhang et al., 2014, 2017; Foster et al., 2016; Ma et al., 2019). Thus, a working hypothesis is that BCP may initially bind to CB2 receptors on midbrain DA neurons and decrease DA neuronal activity or excitability, which may then decrease NAc DA response to METH and subsequent DA-dependent behavior (Figure 7).

To test this hypothesis, we used *in vivo* brain microdialysis to measure extracellular DA in the NAc. We found that systemic administration of METH (1 mg/kg) caused a robust (10-fold) increase in extracellular DA levels in the NAc immediately after administration, which lasted for about 2 h. Pretreatment with BCP, at the same doses that inhibited METH self-administration and reinstatement responding, produced a dose-dependent reduction in METH-enhanced DA release, suggesting that DA-dependent mechanisms may in part underlie BCP’s action against METH (Figure 7).

Notably, BCP alone, at 25 and 50 mg/kg, did not produce a significant alteration in extracellular DA in the NAc, suggesting that it is not rewarding or aversive by itself. This is supported by previous findings that BCP failed to maintain self-administration after substitution for cocaine in rats previously self-administering cocaine (Galaj et al., 2020a) nor produced CPP or conditioned place aversion in mice (Al Mansouri et al., 2014). However, it is slightly different from our previous report that BCP, at higher doses (50, 100 mg/kg), dose-dependently inhibit brain-stimulation reward maintained by either electrical stimulation of the medial forebrain bundle at the lateral hypothalamic level in rats or by optical stimulation of midbrain DA neurons in DAT-Cre mice (He et al., 2020), suggesting that high doses of BCP may be required to produce a significant reduction in NAc DA release. We have previously reported that JWH133, a highly selective CB2 receptor agonist, dose-dependently inhibits cocaine self-administration and decreases NAc DA release, but itself does not produce conditioned place aversion (Xi et al., 2011), suggesting that a reduction in NAc DA release may not necessarily lead to dysphoric or aversive effects. Similarly, aversive stimuli may also increase DA release and individual groups of DA cells make a unique contribution to the processing of reward and aversion (Weele et al., 2019; Verharen et al., 2020), suggesting that multiple neural mechanisms may underlie drug aversion and that BCP’s potentially therapeutic anti-METH effects are unlikely to be mediated by its aversive effects.

In conclusion, BCP is a major component in the essential oils of cannabis and other spice and food plants (Sharma et al., 2016; Galaj and Xi, 2019). In the present study, we demonstrate that systemic administration of BCP is highly effective in attenuating METH-taking and METH-seeking in rodents via both CB2- and non-CB2-dependent mechanisms. Given that BCP is an FDA-approved food additive with good oral bioavailability, favorable pharmacokinetics, and low toxicity, BCP deserves further research as a promising repurposed drug in translational studies for the treatment of METH use disorder.

## Data Availability

The original contributions presented in the study are included in the article/Supplementary Material, further inquiries can be directed to the corresponding author.
